# Reassessing the dynamics between exchange, oil, stock markets and uncertainty during COVID-19 in emerging market economies

**DOI:** 10.1016/j.mex.2022.101990

**Published:** 2022-12-28

**Authors:** Sanjiv Kumar, K.P. Prabheesh

**Affiliations:** Dept. of Liberal Arts, Indian Institute of Technology, Hyderabad, Kandi, Sangareddy, Telangana, India, 502285

**Keywords:** COVID-19, Uncertainty, Exchange rate, Stock prices and oil prices, New Measure of the COVID-19 Pandemic: A New Time-series Dataset

## Abstract

This study reassesses the dynamic relationship between stock, oil, foreign exchange markets, and COVID-19-induced uncertainty. For estimation, we utilize the monthly data from January 2020 to December 2021 and utilize panel vector autoregression econometric techniques in emerging market economies. The empirical findings reveal that heightened uncertainty during the pandemic had a negative impact on stock and oil, and foreign exchange markets. Findings further suggest that uncertainty during the pandemic slanted the relationship between oil and stock and foreign exchange markets due to the precautionary approach followed by economic agents.

• COVID-19 uncertainty negatively impacted the oil, stock, and foreign exchange markets.
• Uncertainty had a non-linear impact on stock and oil prices.
• During the initial period of COVID-19, uncertainty distorted the relationship between oil, stock and exchange markets.

## Graphical abstract

**Figure:**
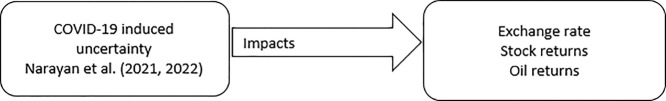

Specifications table

**Table:** 

Subject area:	Economics and Finance
More specific subject area:	Stock markets, oil prices, Exchange rate
Method Name:	New Measure of the COVID-19 Pandemic: A New Time-series Dataset
Name and reference of Original methods:	Narayan, P. K., Iyke, B. N., & Sharma, S. S. [14]. New measures of the COVID-19 pandemic: A new time-series dataset. *Asian Economics Letters*, *2*(2), 23491. https://a-e-l.scholasticahq.com/article/23491-new-measures-of-the-covid-19-pandemic-a-new-time-series-dataset
Resource availability:	NA.

## Method details

The vector autoregression (VAR) was originally proposed by Sims [31] in a time series context which allows for endogenous interaction among variables in the system. The VAR in a panel context was introduced by Holtzeakin et al. [8]. The STATA code was developed by Love and Zicchino [12] and recently modified by Abrigo and Love [1]. These authors have developed PVAR in a generalized method of moments (GMM) framework and employ GMM estimators. Further, this method is effective in controlling the unobserved individual heterogeneity and individual-specific fixed effects. Thus, this method is more advanced in estimating the VAR types of estimation in a panel context; therefore, we employ it for estimation purposes.

A l-variate homogeneous panel VAR of order q with panel-specific fixed effects can be represented in the following equation form:

(1) $$\begin{array}{ccc} Z_{it\;} & = & Z_{it-1\;}B_{1}+\;Z_{it-2\;}B_{2}+\ldots +\;Z_{it-q+1\;}B_{q-1}+\;Z_{it-q}B_{q}+\;D_{it}C+\mu_{i}+\;\varepsilon_{it}\\ & & i\;\varepsilon \;\left\{1,\;2,\;\ldots ,\;n\right\},\;t\;\varepsilon \left\{1,2,\ldots T_{i}\right\} \end{array}$$

Where $Z_{it\;}$ is a (1×l) vector of dependent variables, $D_{it}$ is a (1×m) vector of exogenous covariates. The term $\mu_{i}$ and $\varepsilon_{it}$ are a (1×l) vector of dependent variable-specific fixed-effects and the white noise error term. The (l×l) matrices $B_{1},\;B_{2},\ldots ,\;B_{q-1},\;B_{q}$ and the (m×l) matrix C are the parameters to calculate from estimation. In addition to that, we presume that the innovation term follows the following characteristics: $E(\varepsilon_{it})=0$, $E(\varepsilon_{it}^{\prime }\varepsilon_{it})=\;\Omega$, and $E(\varepsilon_{it}^{\prime }\varepsilon_{iv})=0$ for all t>v.

In order to estimate Eq. (1) with the assumption that errors are serially uncorrelated, by using the difference (FD), it could be consistently estimated by instrumenting legged differences with differences and levels of $Z_{it}$ [2]. However, the FD transformation posed a problem when there is a gap in the unbalanced panel. In order to overcome this issue, Arellano and Bover [4] advocated the forward orthogonal deviation (FOD) transformation, which overcomes the weakness of FD transformation. Eq. (1) can be written in a transformed panel VAR model in a more compact form:

(2) Zit*=⌣Zit*B+eit*

(3) $$Z_{it}^{*}=\;\left[z_{it}^{1*}\;\;z_{it}^{2*},\ldots ,\;\;z_{it}^{p*}\;\;z_{it}^{k*}\right]\;$$

(4) ⌣Zit*=[Zit−1*Zit−2*,…,Zit−q+1*Zit−pk*Wit*]

(5) $$e_{it}^{*}=\left[\;e_{it}^{1*}\;\;e_{it}^{2*}\;,\ldots ,\;e_{it}^{k-1*}\;\;e_{it}^{k*}\right]$$

(6) $$B^{\prime }=\left[B_{1}^{\prime }B_{2}^{\prime },\ldots ,\;B_{p-1}^{\prime }\;B_{p}^{\prime }C^{\prime }\right]$$

Where the asterisk represents the transformation of the original series. Let us assume the original series as $s_{it}$, then the first difference implies $s_{it}^{*}=\;s_{it}-\;s_{it-1}$, whereas forward orthogonal deviation implies that, $s_{it}^{*}=\;\left(s_{it}-\;\bar{s_{it}}\right)\;\sqrt{T_{it}/\left(T_{it}+1\right)},$ where the term $T_{it}$ represent the available future observations for panel i and time t. $\bar{s_{it}}$ represent the mean value of all available future observations.

If we stack observation over the panels rather than over time. The GMM estimator is calculated by

(7) B=(⌣Z*′V⌣WV′⌣Z*)−1(⌣Z*′V⌣WV′Z*)

Where ⌣W represent the (L×L) weighting matrix and follow the characteristics such as nonsingular, symmetric and positive semidefinite and $D_{it}\;\varepsilon \;V_{it}$. By presuming the $E(V^{\prime }e)=0$ and rank E(⌣Zit*′V)=kq+l, then the GMM estimator would be consistent [1].

## Introduction

With the eruption of the COVID-19 pandemic and its subsequent spread of the highly contagious nature of the virus, we saw that many parts of the world economies resorted to adopting unprecedented mitigation measures such as nationwide lockdowns, physical distancing, and restrictions on movements of people domestically and internationally [20]. This resulted in a disruption in the global supply chain, a decline in consumption and a large reduction in economic activities and a disruption in international trade that damaged the global economy [18]. Further, these sudden and unprecedented policy measures taken by governments created an uncertain economic environment for both economic agents and policymakers around global economies. However, during the pandemic, the stock markets reacted distinctively; initially, investors overreacted to the virus, but as the information about the pandemic increased, stock markets subsequently bounced back [20]. But, rising uncertainty and lack of sufficient information about the unfolding of the pandemic and its impact on economic activities and subsequent economic recovery caused uncertainty among policymakers and investors [18].

Furthermore, this increasing uncertainty in directly affected various markets, such as stock markets [7,^10^,^13^,^21^,30] and foreign exchange markets [5,24], Oil prices [9,^21^,29], and international trade [32,33]. Furthermore, due to the sudden lockdown in many parts of the world, oil demand suddenly collapsed^1^, and it went through the highest uncertain time period during the initial phase of the pandemic [6]. Prabheesh et al. [27] argued that oil price movements play a crucial role in foreign exchange and stock markets for oil-importing economies. Thus the present study attempt to address how these dynamics progressed during the pandemic in EMEs.

The theoretical relationship between oil price and exchange rate suggests that a positive shock in oil prices leads to the depreciation of the exchange rates of oil-importing economies and leads to a shift of wealth from oil-importing to oil-exporting economies [28]. For the nexus between oil prices and stock prices, Narayan et al. [16] argued that a positive shock in oil prices leads to an increase in the cost of production in oil-importing economies, thus lower economic growth and causing a decline in stock return. Similarly, the theoretical link between uncertainty and markets implies that an increase in uncertainty causes negative economic growth, which in turn reduces the oil demand and thus reduces the oil prices. In the same way, uncertainty is closely associated with stock and foreign exchange markets in driving their paths [23,26].

There have been studies conducted that analyze the link between uncertainty associated with COVID-19 and its impact on various markets, such as for the Indian economy by Prabheesh and Kumar [23], Prabheesh [22] and for China by Liu (2021) [35] see a survey by Padhan and Prabheesh [18]. It is important to note that it has been more than two years since the outbreak of COVID-19; so far, studies have been conducted based on the preliminary data and are limited to economy-specific and consider only conventional methodology. Thus, there is a lack of studies to analyze the dynamic between oil prices, stock prices and exchange rates and uncertainty by using the large set of emerging economies and employing the new econometric technique.

This study addresses the aforementioned research issues and re-examines the findings of the earlier work by Prabheesh and Kumar [23]^2^. In order to test for the robustness of the findings, we selected 17 EMEs and re-estimated the model. Second, for the estimation purpose, we utilize the panel vector autoregression (PVAR)^3^ given by Love and Zicchino [12] and recently updated by Abrigo and Love [1]. It uses the generalized method of moments (GMM) style for estimation, and for lag selection criterion, it utilizes optimal model and model selection criteria (MMSC) proposed by Andrews and Lu [3]. Using the instruments and fixed effects models improves the efficiency of the model compared to Pedroni [19]; therefore, this method has higher advantages compared to Pedroni structural panel VAR techniques. The empirical findings of the study suggest that due to uncertainty exchange rate depreciated in EMEs. For stock prices, in the initial period, uncertainty had a negative impact but in the later period stock market reacted positively. Similarly, for oil return, in the initial time period, uncertainty negatively impacted the oil return, but over the period, the response was positive.

The study contributes to the literature on the following grounds. First, for a robustness check of the earlier findings, we reassess the dynamic of oil, stock, and foreign exchange markets by taking the large set of EMEs. Second, for estimation purposes, we utilize the PVAR technique, which is relatively new to the literature. Third, we expand the sample period till December 2021, as updated by Narayan et al. [15]. Fourth, from the implication perspective, this study helps policymakers to understand the response of the market to an increase in uncertainty and to design a policy which could be helpful in mitigating the impact of uncertainty on stocks, foreign exchange and oil prices in future.

This study is organized as follows. Section 2 discusses the data, section 3 presents the method, and 4 presents the empirical findings. Finally, section 5 concludes.

## Data

We constructed a balanced panel of 17 EMEs^4^ for the period of January 2020 to December 2021. Data for stock prices and exchange rates are obtained from the CEIC database. The oil prices were proxied by West Texas Intermediate and collected from the US Energy Information Administration's website. Data related to the COVID-19-induced uncertainty index is borrowed from Narayan et al. [14,15]. The authors have constructed five different indices, and an aggregate index is known as the pandemic sentiment index. Among them, an index of uncertainty due to pandemics and epidemics has been utilized for the estimation. This uncertainty index captures the uncertainty related to the pandemic by utilizing the 45 most popular newspapers worldwide and using the 327 keywords related to a different aspect of COVID-19. Thus, this uncertainty index is superior to the other existing uncertainty index used in the literature [13]. For estimation, we have converted the oil prices and stock prices into returns based on the literature^5^.

## Methods

For estimation, we employ the PVAR model, which combines the traditional VAR model and allows for endogenous interaction among variables in the system and also helps in controlling unobserved individual economy heterogeneity [1]. In order to capture the impact of uncertainty on the oil return, exchange rate and stock return, we write the empirical equation in the following form:

(8) $$Y_{it}=\left[UI_{t},OR_{t},ER_{it},SR_{it}\right]$$

Where $Y_{it}$ represent the endogenous variables, $OR_{t}\;$represent the oil return, ER represent the exchange rate, SR represents the stock return, and UI represents the uncertainty index. The reduced form dynamic relationship among selected variables is as follows:

(9) $$\begin{array}{ccc} Y_{it} & = & A_{0i}+\;A_{1}\left(l\right)Y_{it-1}+\varepsilon_{it,}\\ & & i\;\in \left\{1,\;2,\ldots ,\;22\right\},\;\;t\;\in \left\{1,\;2,\ldots 16\right\} \end{array}$$

Where $A_{0i}$ is a vector of time-invariant country-specific intercepts, $A_{1}\left(l\right)$ are 4×4 matrices of lagged coefficient that include the own and cross-section effects of the lag of the endogenous variable on their current observations. The term *ɛ_it_* represent a (4×1) vector of idiosyncratic disturbance. The study also presumes that innovation follows the ensuing properties: E ($e_{it})=0,\;E\left(e_{it}^{\prime }e_{it}\right)=\Sigma ,$ and $E(e_{it}^{\prime }e_{iv})=0$ for all t > v.

The PVAR model is estimated by utilizing the GMM estimator as provided by Abrigo and Love [1]. In order to estimate the impulse response function and forecast error variance decomposition (FEVD), we impose restrictions. We presume that the UI is given to EMEs; thus, it is presumed to be exogenous to the system, similar to [34]. Second, oil prices are endogenous to UI but exogenous to all other variables in the system. The variable ER is endogenous to UI and OR and exogenous to SR. Finally, we presume that SR is most contemporaneously endogenous to all the variables in the system. For ordering the variables, we followed Prabheesh and Kumar [23].

## Discussion of results

**Fig. 1:**
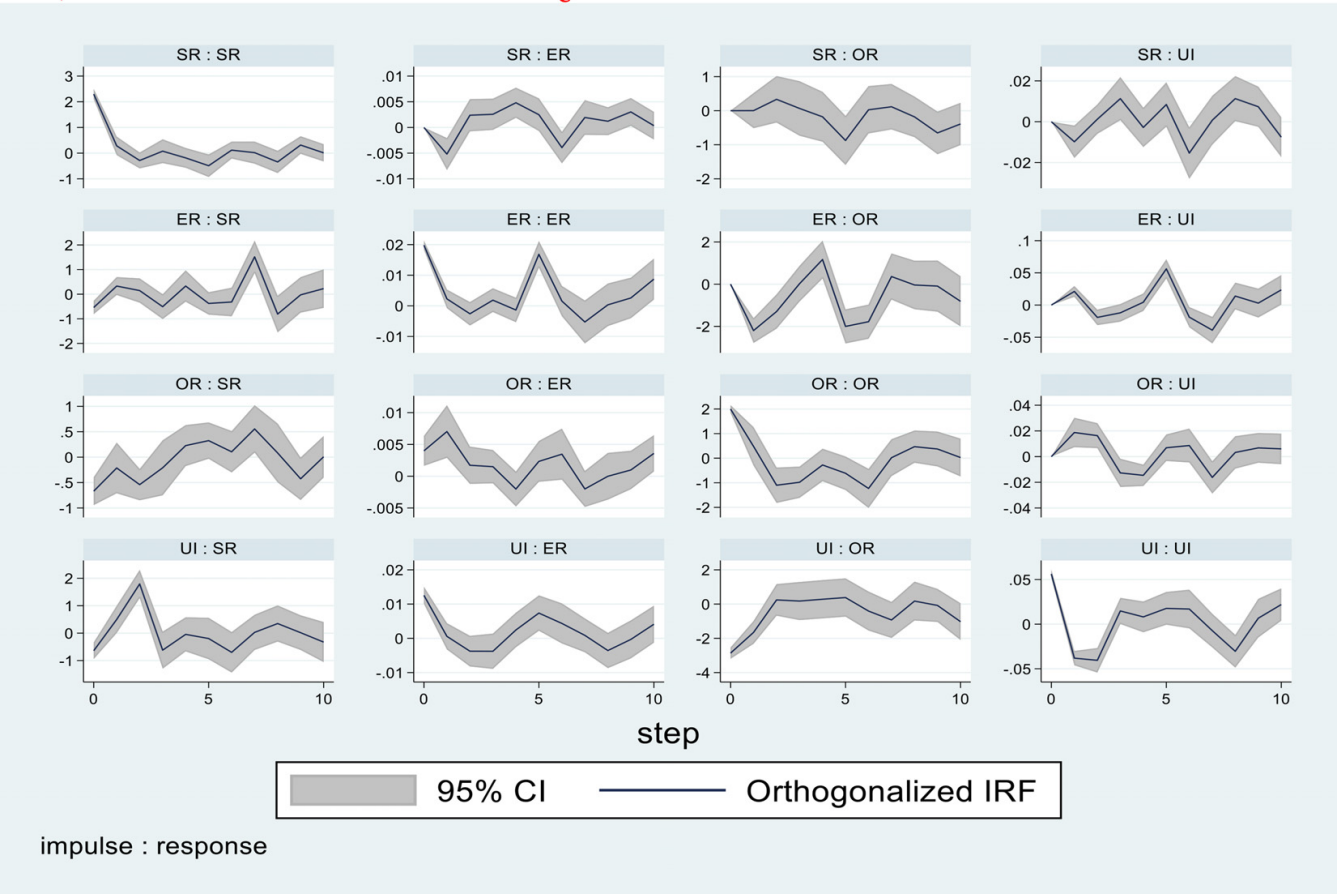
Impulse Response Function, This figure displays the impulse response function estimated by using the PVAR model. The PVAR model includes UI, OR ER and SR. The model is estimated using a 95% confidence interval.

Source: Authors' calculation

Initially, we check the stationarity properties and stability condition of the selected PVAR model. The variables OR, SR, and UI are found to be level stationary, and ER is found to be difference stationary^6^. Fig. 1 presents the impulse response function of the estimated model. The first row of the figure depicts the response of ER to positive shock in SR is found to be negative and significant (SR: ER). It implies that during the pandemic, when SR increases, their currency is appreciated. These findings are in line with the theoretical expectation as the stock return increases, in response to higher stock return, capital flows take place and which leads to currency appreciation. The second row represents the response of SR to a positive shock in the ER is found to be negative in the first month. It indicates that in the first month of the pandemic, as the exchange rate appreciated, the response of the stock market was negative. These findings are opposite the theoretical expectation. From there, it can be inferred that during the COVID-19 pandemic period, when ER appreciated, the SR responded negatively. These findings indicate that during the initial month of the pandemic, the relationship between ER and SR slanted; these findings are in line with [23,25]. Moreover, from the second month onward, a positive shock in the ER is associated with an increase in the SR.

The third row of the figure presents a positive shock in the OR and the response of the other variables in the system. For instance, the response of SR to a positive shock in OR is found to be negative and significant (OR: SR). It implies that an increase in oil prices negatively impacted the stock markets in oil-importing countries^7^. These findings are in line with theoretical expectations; an increase in oil prices raises the cost of production and reduces economic growth leading to a reduction in stock prices (Narayan et al., 2014, [17]). Similarly, the response of the ER to a positive shock in the OR is found to be positive and significant. It implies that during the pandemic, when the oil prices increased, it negatively impacted the exchange rate in EMEs. These findings are again in line with theoretical expectations; a rise in oil prices leads to the depreciation of currencies of oil-importing economies [28].

From the last row, it can be observed that in the first month, a shock in the UI is associated with a negative response of SR (UI: SR). It implies that an increase in uncertainty negatively affected the SR in EMEs. Also, after the second month onwards, the response of SR was positive and significant. It indicates that from the second month onwards, the stock market reacted positively. Our empirical findings are in line with Phan and Narayan [20], who argued that during the initial phase of the pandemic, stock markets reacted adversely, but over the period, the response of stock markets was positive. Similarly, Liu et al. [11], from a time-varying perspective, found that in the later period, pandemics had a positive impact on stock markets.

The response of ER to a positive shock in the UI is found to positive and significant. It implies that an increase in uncertainty during the pandemic depreciated the exchange rate in EMEs. These findings are in line with the theory; that when there is uncertainty in the global economy, a capital flight takes place for safety from EMEs to advanced economies. Thus, it shows that when uncertainty increased during the pandemic, the ER depreciated. Likewise, a shock in the UI is also associated with a negative and significant response to OR. It implies that during the pandemic increase in uncertainty negatively impacted the oil return. These findings are in line with earlier studies such as Narayan and Devpura [6], who found that initially, COVID-19 impacted the oil market adversely.

Overall our study's findings align with earlier studies' findings by Prabheesh and Kumar [23] and confirm that a positive shock in uncertainty negatively impacted the oil return and exchange rate. However, the response of the stock market to uncertainty was peculiar; during the initial period, the stock market reacted negatively, but from the second month onward response was positive. Further, empirical findings suggest that during the initial phase of the pandemic, the relationship between OR, SR and ER was distorted due to prevailing heightened uncertainty. However, as the flow of information increased about the pandemic, and other medical advancements, such as the development of vaccines, the market behaved in line with expectations. Table 1 presents the forecast error variance decomposition (FEVD) of OR, ER and SR. In the case of oil return, in the 5^th^ month, the variation in innovation explained by UI is 43.3%. It indicates that during the pandemic, uncertainty significantly explained the variation in the stock return. Similarly, in the case of ER and SR, in the 5^th^ month, the variation in innovation explained by the uncertainty is about 26% points and 37% points, respectively. It implies that during the pandemic, uncertainty significantly explains the variation in the exchange rates and stock return. These findings suggest that during the pandemic, uncertainty significantly explains the variation in the innovation of ER and SR in EMEs. It also significantly explains the variations in the case of oil return. For sensitivity analysis, we utilize the volatility index (VIX) as an alternative proxy variable for uncertainty and re-examine the PVAR. The overall responses are similar to our main findings^8^. Findings are not reported due to space constraints but are available from the authors.

**Table 1:** Forecast Error Variance Decomposition, This table exhibits the forecast error variance decomposition. Where OR represent oil return, ER is the exchange rate, SR is stock return, UI is the uncertainty index, and column 1 represents the months.

Months	UI	OR	ER	SR
OR				
1	0.672	0.327	0	0
5	0.433	0.252	0.307	0.005
10	0.327	0.231	0.403	0.036
ER				
1	0.278	0.028	0.693	0
5	0.260	0.101	0.554	0.084
10	0.231	0. 080	0.605	0.081
SR				
1	0.063	0.069	0.045	0
5	0.373	0.077	0.069	0.480
10	0.300	0.091	0.243	0.364

Source: Authors' calculation

## Conclusion

COVID-19 induces uncertainty had a devastating impact on various markets all around the world. There have been many studies done based on the initial availability of the dataset, but their robustness testing remained. Thus, this study re-examines the dynamic relationship between the three most important markets, such as the exchange rate, the oil market and the stock market, in the prevalence of COVID-19-induced uncertainties from an EMEs perspective. Our findings reaffirm that uncertainty associated with COVID-19 had dampened the oil return and negatively impacted the SR and ER also depreciated in emerging economies. Further, the study reinforces that initially, due to heightened uncertainty relationship between oil, stock and foreign exchange markets was distorted.

## Funding Source Declarations

The authors declare that no funding has been received to carry out this research work.

## Declaration of Competing Interest

This article is submitted to MethodsX as a special issue in COVID Research Robustness. The authors declare that they have no known competing financial interests or personal relationships that could have appeared to influence the work reported in this paper.

## Figures and Tables

**Table 1A:** Variables details.

Variable	Short name	Measurement	Source
SR	Stock return	Log($SP_{t}/SP_{t-1})*100$	CEIC database
OR	Oil retun	Log($OP_{t}/OP_{t-1})*100$	https://fred.stlouisfed.org/series/DCOILWTICO
ER	Exchange Rate	Log(ER)	CEIC database
UI	Uncertainty Index	Uncertainty index	Narayan et al. [15]
VIX	Volatility index	Volatility index	https://fred.stlouisfed.org/series/VIXCLS

Source: Authors’ compilation

## Data Availability

Data will be made available on request.

## Appendix A

Table 1A.

## Footnotes

1 In April 20, 2020, oil price traded in negative territory [18].

2 Prabheesh and Kumar [23], examine the impact of COVID-19-induced uncertainty on stock and foreign exchange in the case of the Indian economy only.

3 There are two existing panel vector autoregression (PVAR). one proposed by the Padroni [19] and other given by the Love and Zicchino [12] and recently modified by Abrigo and Lobe [1].

4 The selected 17 EMEs are as follows Argentina, Brazil, Bulgaria, Chile, Colombia, Hungary, India, Indonesia, Malaysia, Mexico, Morocco, Philippines, Peru, Poland, Romania, Thailand, and Turkey.

5 For more information, see Prabheesh and Kumar [23]

6 The optimal lag is found to be 6, and at that lag the model is found to be stable, as all the eigenvalues lie inside the unit circle. Results for lag selection, stability and unit root test are not reported due to space constraint but available from the authors.

7 As our sample economies includes the oil importing emerging market economies.

8 From the impulse response function, the uncertainty data from the Narayan et al [14,15] has more significant impact compared the volatility index data obtained from CBOE volatility index.
